# Embryonal Rhabdomyosarcoma of the Biliary Tree as a Differential in a Paediatric Patient Presenting with Biliary Dilatation: Not Always a Choledochal Cyst

**DOI:** 10.15388/Amed.2021.29.1.2

**Published:** 2022-01-24

**Authors:** Tara Prasad Tripathy, Yashwant Patidar, Karamvir Chandel, Annapoorani Varadarajan, Vikrant Sood, Shalini Thapar Laroia

**Affiliations:** Department of Radiodiagnosis, AIIMS, Bhubaneswar, India; Department of Interventional Radiology, Institute of Liver & Biliary Sciences; D-1 Vasant Kunj, New Delhi-110070, India; Department of Intervention Radiology, Institute of Liver & Biliary Sciences; D-1 Vasant Kunj, New Delhi-110070, India; Department of pathology, Institute of Liver & Biliary Sciences; D-1 Vasant Kunj, New Delhi-110070, India; Department of Pediatric hepatology, Institute of Liver & Biliary Sciences; D-1 Vasant Kunj, New Delhi-110070, India; Department of Radiology, Institute of Liver & Biliary Sciences; D-1 Vasant Kunj, New Delhi-110070, India

**Keywords:** biliary rhabdomyosarcoma, choledochal cyst, diffusion weighted imaging, tulžies rabdomiosarkoma, tulžies pūslės akmenys, difuzijos svertinis tyrimas

## Abstract

Rhabdomyosarcoma is a soft tissue malignant musculoskeletal tumour and is the most prevalent soft-tissue sarcoma in the paediatric population. Although, Embryonal RMS of the biliary tree is a rare entity, however, it is the most common cause of paediatric malignant obstructive jaundice. We present a 4-year-old child who had symptoms of obstructive jaundice and palpable liver. The non-contrast magnetic resonance imaging and magnetic resonance cholangiopancreatography (MRCP) features were consistent with choledochal cyst. However, contrast enhanced computed tomography and PET CT images revealed biliary RMS as the differential diagnosis. Percutaneous biopsy followed by histopathology confirmed the diagnosis of embryonal biliary RMS. Since embryonal rhabdomyosarcoma is uncommonly recorded in the literature and can mimic the appearance of a choledochal cyst, this case report emphasises the necessity of keeping embryonal RMS as a differential in paediatric cases of obstructive jaundice.

## Santrauka.

Rabdomiosarkoma yra piktybinis raumenų ir skeleto minkštųjų audinių navikas; tai – dažniausiai pasireiškianti minkštųjų audinių sarkoma vaikams. Nors bendrojo tulžies latako embrioninė rabdomiosarkoma pasitaiko retai, tačiau ji yra dažniausia vaikų piktybinės obstrukcinės geltos priežastis. Aprašome 4 metų vaiko, kuriam pasireiškė obstrukcinės geltos simptomai ir kurio kepenis buvo galima apčiuopti, atvejį. Magnetinio rezonanso tomografija nenaudojant kontrastinio skysčio ir magnetinio rezonanso cholangiopankreatografija (MRCP) rodė tulžies pūslės akmenligę. Tačiau kompiuterinės tomografijos naudojant kontrastinį skystį ir PET/KT tyrimai parodė, kad gali būti svarstoma tulžies rabdomiosarkomos diagnozė. Atlikus perkutaninę biopsiją, o paskui histopatologinį tyrimą, patvirtinta embrioninė tulžies rabdomiosarkoma. Embrioninė rabdomiosarkoma retai aprašoma literatūroje ir gali atrodyti kaip tulžies pūslės akmenys. Aprašant šį atvejį siekiama, kad būtų svarstoma embrioninės rabdomiosarkomos diagnozė tais atvejais, kai vaikams pasireiškia obstrukcinė gelta.

## Introduction

Rhabdomyosarcoma (RMS) is a malignant tumour that most commonly affects children’s head and neck, genitourinary system, and limbs [1,2,3]. Embryonal RMS of biliary origin is a rare occurrence, with only a few reports in the literature, the biggest of which was a 25-patient series [2]. Due to its low incidence, strong suspicion on imaging plays a crucial role in diagnosis [3]. This case report describes multimodality imaging findings and justifies the inclusion of biliary RMS as a differential in a child with obstructive jaundice.

## Case report

**Figure 1.**
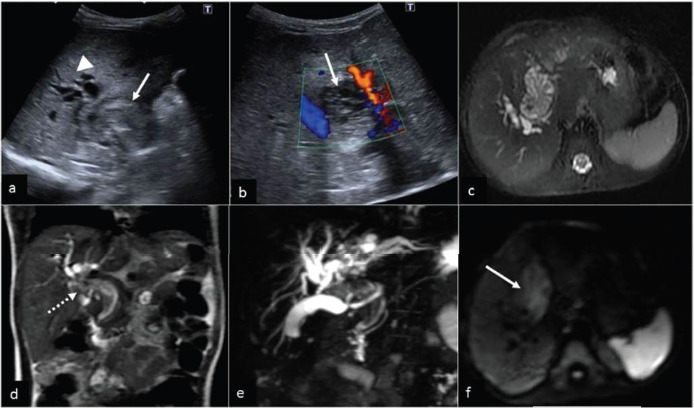
(a) US showing heteroechoic intraductal portal mass (white arrow) causing bilobar IHBRD (arrow-head) and (b) no definite blood flow within the mass on Colour Doppler. (c) Coronal T2-weighted magnetic resonance imaging illustrating a heterogenous predominantly hyperintense lobulated-shaped mass arising within the common bile duct (white dashed arrow,) extending superiorly to the level of the porta. (d) oronal magnetic resonance cholangiopancreatography shows large filling defect with dilated intrahepatic bile ducts. (e) DWI (b=800s/mm^2^) (image c) shows mild restricted diffusion in mass (white arrow).

Following a brief episode of fever, a 4-year-old child arrived with a 2-month history of yellow staining of the eyes, high-coloured urine, pale faeces, and developing abdominal distension. Elevated liver enzymes (AST/ALT/ALP/GGT: 357/190/719/743 IU/ml) along with conjugated hyperbilirubinemia (T. bilirubin: 5.8, d. bilirubin: 2.8 mg/dl) were noted on admission. Abdominal ultrasound (US) revealed a heterogeneous intraductal mass (4.3×2.3×3.4cm) producing dilatation of the common bile duct (CBD) and intrahepatic biliary channels with no evident internal flow on colour Doppler (Figures 1a and 1b).

**Figure 2.**
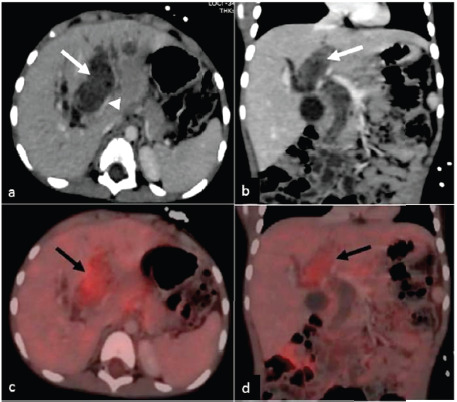
Post contrast computed tomography axial (a) and coronal (b) image demonstrating a mild enhancing heterogeneous mass within the common bile duct and left hepatic duct(arrow) causing compression of left portal vein seen(arrowhead). Axial (c) and coronal (d) PET CT showing mild metabolic activity within the soft tissue mass (black arrow).

Magnetic resonance cholangiopancreatography (MRCP) revealed a lobulated lesion involving the CBD and left hepatic duct along with fusiform dilatation of the CBD. (Figure 1 c, d, e.) Diffusion restriction was noted (Figure 1f). Possibility of a choledochal cyst with luminal cast was considered with possibility of intraluminal mass as differential. A contrast enhanced computed tomography (CECT) scan of the abdomen revealed a hypodense intraductal fusiform-shaped mass (65 mm × 45 mm × 30 mm) running along the length of the left hepatic duct (LHD), CBD, up to secondary biliary confluence, with slight heterogeneous enhancement (Figure 2a, b). There was some attenuation of the left portal vein, with no signs of tumour thrombosis. Intrahepatic bile ducts were dilated. GB was distended. There was no ascites. Periportal, peripancreatic and portocaval lymph nodes were enlarged.

**Figure 3.**
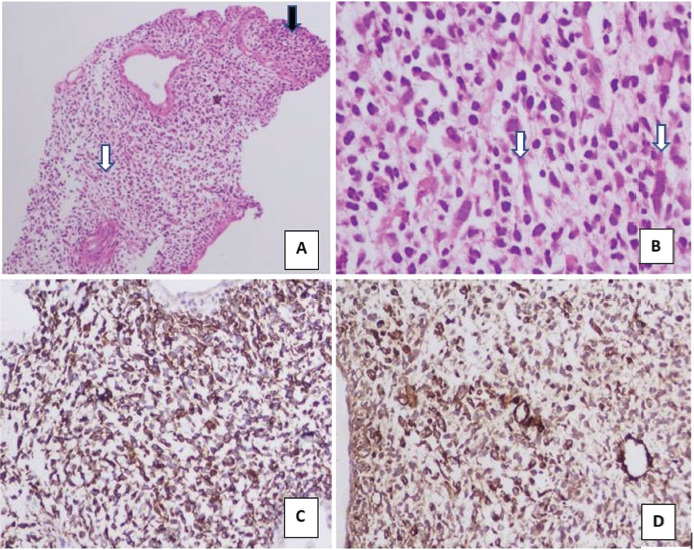
**Intraductal biopsy**: Section shows a variably cellular tumour revealing. **A)** Hypocellular (white arrow) and hypercellular areas (Black arrow) with sheets of small rounded to spindle cells having inconspicuous nucleoli and scant cytoplasm. Hypocellular area reveals scattered tumour cells in a myxoid stroma – *(Haematoxylin & Eosin, 4x).* **B)** Few of the scattered tumour cells have abundant elongated eosinophilic cytoplasm (tadpole cells – white arrows). Dense condensation of tumour cells forming cambium layer was also seen (not shown). No significant anaplasia was seen in section examined – *(Haematoxylin & Eosin, 40x).* On immunohistochemistry, the tumour cells show **C)** positive for desmin *(Desmin Immunohistochemistry, 10X)* and **D)** positive for Smooth Muscle Actin (SMA) *(SMA Immunohistochemistry, 10X)*

US guided percutaneous punch biopsy from the lesion was done. Microscopy confirmed the presence of a variably cellular tumour consisting of hypocellular and hypercellular showing primitive mesenchymal cells of varying phases of myogenesis, sheets of small, rounded to spindled cells with inconspicuous nucleoli and scant cytoplasm (rhabdomyoblasts/Tadpole cells) (Figure 3 a, b). Immunohistochemical test was suggestive of embryonal RMS with positive stains of desmin and smooth muscle antigen (Figure 3 c, d). Dense zone of tumour cells forming cambium layer was also evident without significant anaplasia.

Positron emission tomography (PET) scan demonstrated metabolically active soft tissue density content within the common bile duct (see Figures 2 c, d.). Metabolically active peripancreatic, periportal, portocaval lymph nodes were noted. There were no signs of distant metastases.

Final diagnosis of embryonal RMS of biliary tract stage T1, N1 (with regional lymph node metastases), M0 (no distant metastases) was made. Patient was planned for neoadjuvant chemotherapy as per international RMS study group.

## Discussion

The most common soft-tissue sarcoma in children is RMS, which accounts for 5–10% of all malignant solid tumours [1]. RMS is the fourth most common childhood tumour, following central nervous system tumours, neuroblastomas, and nephroblastomas [1]. The biliary tree is involved in only 0.5% of RMS cases [3]. The ICR (International Classification of RMS) is a prognosis-based classification system based on histological subtypes [better prognosis (botryoid and spindle cell RMS), intermediate prognosis (typical embryonal RMS), and poor prognosis (alveolar RMS)] [4]. In the paediatric population, the embryonal and alveolar variants of RMS are the two most common histological subtypes. The embryonal RMS accounts for the vast majority of cases (almost 70%) and typically affects children under the age of eight [1,5]. Myoblastic differentiation and expression of skeletal muscle markers such as desmin, myogenin, and/or myoD1 are histological features of RMS [5].

The average age of presentation is three years, with a slight male predominance [6]. The most common clinical manifestation is obstructive jaundice (60–80%) and can be accompanied by hepatomegaly, abdominal distension, and acholic stools [6]. Less common symptoms include pain, nausea, vomiting, and fever [2]. Elevated liver enzymes and conjugated bilirubinaemia are noted [3]. Choledochal cysts, choledocholithiasis, chronic cholangitis strictures, and unusual neoplasms such as biliary RMS can all cause obstructive jaundice in children after the neonatal period [3]. Other than embryonal RMS, there are no neoplasms that develop from the bile ducts in children [6].

Abdominal ultrasonography is frequently the first imaging examination performed in individuals with obstructive jaundice. A soft-tissue mass in the vicinity of the porta hepatis was seen in our case, along with a nearby mass effect and intrahepatic biliary dilatation. In our patient, magnetic resonance imaging and MRCP was done initially which revealed dilated biliary system with casts/luminal sludge. However, on retrograde evaluation the casts were isointense to muscle on T1-weighted imaging and was hyperintense on T2WI. Diffusion weighted imaging shows restricted diffusion which is a clue for diagnosing embryonal RMS. So, we propose that DWI be included in all MRI protocols of paediatric jaundice evaluation. Larger lesions of biliary RMS present with haemorrhage and necrosis and the imaging features of necrosis may mimic the appearance of a choledochal cyst [3]. CECT confirmed that intraluminal contents were mass rather than casts or sludge.

Biliary RMS is often misdiagnosed as a choledochal cyst on imaging which was also in our case with non-contrast MR as the first cross-sectional imaging modality [3]. However, demonstration of diffusion restriction on DWI, enhancement of intraductal material within the biliary tree on CECT, metabolically active intraluminal biliary contents were suggestive of biliary RMS [3]. The presence of arterial waveforms within the intraductal mass on US Doppler is also another clue, however, in our case this feature was not seen [7]. The presence of localised lymph nodal involvement or distant metastases on imaging adds to the likelihood that the obstructive jaundice is caused by a neoplasm [3].

The current management consists of multimodality management including surgical removal, radiotherapy and chemotherapy [2]. Neo-adjuvant chemotherapy followed by resection of the residual tumour has been associated with favourable outcomes [3].

Biliary RMS is an uncommon condition that should be considered in the differential diagnosis of any child who has obstructive jaundice with choledochal cyst on imaging findings. The demonstration of enhancement of intraductal material within the biliary tree, diffusion restriction and the presence of arterial waveforms within the intraductal mass assists in the diagnosis of biliary RMS.
